# Coeliac Disease Pathogenesis: The Uncertainties of a Well-Known Immune Mediated Disorder

**DOI:** 10.3389/fimmu.2020.01374

**Published:** 2020-07-08

**Authors:** Margaret R. Dunne, Greg Byrne, Fernando G. Chirdo, Conleth Feighery

**Affiliations:** ^1^Department of Surgery, Trinity Translational Medicine Institute, Trinity College Dublin, St. James's Hospital, Dublin, Ireland; ^2^School of Biological & Health Sciences, Technological University, Dublin, Ireland; ^3^Instituto de Estudios Inmunologicos y Fisiopatologicos - IIFP (UNLP-CONICET), National University of La Plata, La Plata, Argentina; ^4^Department of Immunology, Trinity College Dublin and St. James's Hospital, Dublin, Ireland

**Keywords:** coeliac disease, pathogenesis, enteropathy, immunopathology, innate and adaptive immune response, molecular mechanisms of disease

## Abstract

Coeliac disease is a common small bowel enteropathy arising in genetically predisposed individuals and caused by ingestion of gluten in the diet. Great advances have been made in understanding the role of the adaptive immune system in response to gluten peptides. Despite detailed knowledge of these adaptive immune mechanisms, the complete series of pathogenic events responsible for development of the tissue lesion remains less certain. This review contributes to the field by discussing additional mechanisms which may also contribute to pathogenesis. These include the production of cytokines such as interleukin-15 by intestinal epithelial cells and local antigen presenting cells as a pivotal event in the disease process. A subset of unconventional T cells called gamma/delta T cells are also persistently expanded in the coeliac disease (CD) small intestinal epithelium and recent analysis has shown that these cells contribute to pathogenic inflammation. Other unconventional T cell subsets may play a local immunoregulatory role and require further study. It has also been suggested that, in addition to activation of pathogenic T helper cells by gluten peptides, other peptides may directly interact with the intestinal mucosa, further contributing to the disease process. We also discuss how myofibroblasts, a major source of tissue transglutaminase and metalloproteases, may play a key role in intestinal tissue remodeling. Contribution of each of these factors to pathogenesis is discussed to enhance our view of this complex disorder and to contribute to a wider understanding of chronic immune-mediated disease.

## Introduction

Coeliac disease (CD) is a common inflammatory disorder of the small intestine resulting in malabsorption. The seminal breakthrough was the discovery by Willem Dicke in 1950 that the wheat protein gluten was the essential trigger causing the disease (1). In the following decades, debate continued about the pathogenic mechanisms involved. Various theories were considered, including the concept that gluten caused direct toxic damage to the intestine, that an enzyme deficiency resulted in failure of gluten degradation or that gluten activated the immune system, driving consequential gut damage (2).

Gluten-induced activation of the adaptive immune response has now been described in great detail and a central role for immune system involvement is widely accepted. A key finding supporting the role of the adaptive immune response was the discovery that certain MHC class II molecules, in particular HLA-DQ2, were a critical requirement for the development of CD (3, 4). It was subsequently demonstrated that gluten-derived peptides bound avidly to these MHC class II molecules, enabling T helper (T_H_) cell activation (5, 6). The binding of these peptides was markedly enhanced following their modification by the enzyme tissue transglutaminase (TG2) (7). The role of TG2 was in part a serendipitous discovery, following the finding that specific IgA antibodies found in CD were directed against this enzyme (8).

Although initially considered a homogenous disorder, it is now recognized that CD encompasses a wider clinical spectrum. Patients can report a range of symptoms, some with clear evidence of malabsorption and others virtually asymptomatic (9, 10). Following treatment with a gluten-free diet, some patients become acutely and rapidly sensitive to accidental gluten exposure, whereas others may show little evidence of a reaction (11–13). A pediatric presentation was the early classic form of CD but it is now accepted that the condition can present at any age, even into the seventh and eighth decades (14). A true increasing incidence of CD is also reported, presumably reflecting a range of yet to be identified environmental triggers of the condition (15). The histological lesion can also vary considerably, with some patients having only a minimal lymphocyte infiltration of the gut epithelium (16), while others display profound changes, so called villous atrophy (17, 18). The term “potential coeliac disease” is also used for people with normal small intestinal mucosa who are at increased risk of developing CD as indicated by positive coeliac serology (10). Malignancy is the final outcome in a subset of patients, but fortunately develops only in a minority of subjects (9). The common type of malignancy associated with CD, enteropathy-associated T cell lymphoma (EATL), is lymphocytic in origin, reflecting the profound dysregulating effects gluten has on the local immune system.

CD is sometimes considered to be an auto-immune disorder and certain features support this contention, including a female predisposition, HLA association, and comorbidity with many classic autoimmune diseases, in particular thyroid disease (e.g., Grave's disease, Hashimoto's thyroiditis) and insulin dependent diabetes mellitus (19). As in other auto-immune conditions, a contribution by the intestinal microbiome is postulated (20). However, if CD is caused by autoimmunity, one feature makes this pathology unique: exclusion of an exogenous trigger i.e., gluten from the diet, causes remission of symptoms and mucosal damage. Nonetheless, some doubt continues as to whether gluten exclusion fully restores a normal small intestinal mucosa or whether consequences of the previous strong immune activation remain. It has long been observed that chronic immune activation by gluten in CD patients induces a permanent change in the intra-epithelial lymphocyte (IEL) compartment, characterized by an expanded and persistent presence of γ/δ IELs, which was recently verified using cell sequencing methodology (21–23). This observation suggests that even in apparently healthy tissue, some subtle changes persist. However, such findings are difficult to interpret given that many patients ostensibly on a gluten-free diet continue to consume trace amounts of gluten (24).

Irrespective of the above variables, a constant feature in all patients is the MHC class II association, with virtually all displaying a HLA-DQ2 or HLA-DQ8 genotype (25). As many as 39 other non-HLA loci have been found to associate with CD and their potential contribution to disease heterogeneity has yet to be determined. Remarkably, many non-coding regions are located in these loci and their potential regulatory effects have just started to be revealed (26). In addition, epigenetic factors may modulate disease risk (27).

In addition to the well-established evidence of the adaptive immune response to gluten, leading to damage to the intestine, in this review we consider the possible involvement of other immune components. Of note, it has been reported that activation of the innate immune response may be a pre-requisite for gluten stimulation of the adaptive response (28–30). The α-gliadin peptide, p31-43, claimed to be responsible for activation of this innate response, was also reported to cause direct damage to the CD mucosa (28). Many studies report that innate immune components such as neutrophils (31), eosinophils (32–34), mast cells (35, 36) and complement proteins (37) are activated in the disease process and potentially contribute to disease pathogenesis. Consideration of this information may lead to a more comprehensive understanding of CD pathology.

## How Does the Lesion Develop in CD?

Despite the series of seminal discoveries made concerning activation of the adaptive immune system in CD, the precise mechanisms responsible for development of the lesion remain uncertain. Specifically, what events cause the tall small intestinal villi to take on a flattened appearance in which villi are either entirely absent or stubby in appearance? It is evident from both *in vivo* and *in vitro* studies that enterocyte damage happens rapidly following gluten exposure (38–43). Yet the question remains, how does this lead to the eventual pathological features of the lesion? Interestingly, although enterocytes are targeted in CD, there is no evidence of tissue necrosis or ulceration, as is observed in small intestinal Crohn's disease (44). Although it is evident that lymphocytes closely located to enterocytes display cytotoxic properties (45, 46), is lymphocyte cytotoxicity the exclusive or principal mechanism responsible for the tissue lesion in CD?

## The Histological Lesion in CD

Biopsy of the small intestine is still the gold standard diagnostic test in the investigation of CD. The lesion can display a range of abnormalities and Marsh proposed a grading system, subsequently modified by Oberhuber et al. (18), which is now commonly used. The Marsh I lesion is characterized by an almost normal mucosa except for the infiltration of villi by IELs, the Marsh II lesion by the additional presence of crypt hypertrophy, and the Marsh III lesion by flattening of the mucosa caused by so-called villous atrophy and swelling of the *lamina propria*. Although an increase in IELs is observed in all CD biopsies, in some patients this increase may be limited to the tip of villi (16, 47); even in the presence of this minimal lesion, some display typical clinical features of CD including malabsorption. Paradoxically, in other patients with a Marsh I lesion, there may be no apparent evidence of malabsorption: these include patients with dermatitis herpetiformis (48), first degree relatives of CD patients and individuals with potential CD (10).

Although villous atrophy and infiltration of IELs are the major reported features in coeliac mucosa, this is based on the limited information provided by standard tissue staining and the two-dimensional image observed with the light microscope. More details can be provided by additional staining of further cell populations and other structures. One feature of the remodeled mucosa is alteration in the microvasculature and these immature vascular structures may result in increased vessel permeability, allowing cells and molecules access to the tissue (49). It has been proposed that improved understanding of the true nature of the CD lesion could be deduced by 3-D printing and computerized modeling of the tissue (50). In recent times, robust flow cytometry methodology and quantification of gamma/delta (γδ) T cells have been proposed as complementary methods for aiding CD diagnosis and monitoring, particularly helpful in resolution of more difficult clinical cases (51, 52). Analysis of an increase in γδ^+^ T cells with a decrease in CD3^−^ IEL, the so-called “coeliac lymphogram,” was also shown to be useful in diagnosing seronegative CD cases (53). Such novel methods have proven superior to traditional serological monitoring methods.

## The Enterocyte—A Target Cell in CD?

Abnormalities in the morphology of enterocytes are usually present in CD but are rarely commented on in routine histology reports. These changes include a reduction in cell height with the cell assuming a cuboidal instead of the normal columnar shape and the migration of the nucleus from the typically basal to a more apical position (54). Using a high content Cellomics analysis system, we studied enterocyte morphology in detail and confirmed a reduction in enterocyte height and noted shape changes in the nucleus (55). Importantly, these changes were found not only in patients with active CD but also in patients with potential CD. Changes in the microvilli at the enterocyte apex may also be noted, even when examined by light microscopy, although these features are more clearly evident when studied by electron microscopy (56). Microvilli are found to be either sparse, absent or have irregular shapes.

These abnormal enterocyte features are presumably caused by the inflammatory response in CD. The changes may preface cell death, and indeed, increased small intestinal enterocyte apoptosis has been demonstrated in CD in several studies (40, 57, 58). Using TUNEL staining to detect fragmented DNA, Moss et al. reported this finding in patients with untreated CD, and evidence of apoptosis correlated with the level of enterocyte proliferation (57). Maiuri et al. also described increased apoptosis in CD tissue but these abnormalities appeared confined to tissue areas displaying evidence of damage (40). Another study reported increased expression of both FAS and FAS ligand death receptors in the duodenal epithelium in untreated CD patients, together with increased perforin expression and number of TUNEL positive cells (59).

Raised circulating levels of intestinal fatty acid-binding protein (I-FABP) in patients with active CD also provides evidence of enterocyte damage (60–62). I-FABP is a low molecular weight protein, specific to small intestinal epithelial cells. Since I-FABP is highly expressed in the cytoplasm of these cells, the circulating level of I-FABP is a very sensitive marker for monitoring enterocyte damage and has been proposed as a potential biomarker of disease activity in CD (63). Interestingly, in patients with severe enteropathy, strong expression of I-FABP is also noted in the crypts, and this may be linked to an accelerated developmental program of enterocyte proliferation and differentiation. As a consequence, while I-FABP is expressed in fully differentiated enterocytes in homeostasis, it appears earlier in crypt enterocytes when enteropathy is present (64).

## A Broader Role for Intestinal Epithelial Cells?

Enterocytes are the predominant intestinal epithelial cell type and together with other cells, including goblet cells, Paneth cells and M cells, act as a first line of defense against potential access from the gut of microorganisms and other noxious agents (65, 66). However, intestinal epithelial cells have a wider role in gut homeostasis, interacting on a constant basis with commensal organisms and influencing the behavior of cells of both the innate and adaptive immune system. Reactions with microogranisms and danger signals is facilitated by the epithelial surface expression of a range of innate receptors including toll like receptors (67). Epithelial cells influence the behavior of many intestinal cell populations including innate lymphoid cells (ILC), neutrophils, basophils, macrophages, T cells and B cells through the production and release of a range of cytokines and chemokines including tumor necrosis factor alpha (TNF)-α, interleukin (IL)-8, IL-18, IL-25, transforming growth factor (TGF)-β and B cell activating factor (66, 68). Amongst its many roles, a key function of intestinal epithelial cells is to allow the orderly paracellular absorption of nutrients and ions and to prevent access to potentially damaging substances including dietary antigens. This led to the study of a series of tight junction structures and the discovery of the protein zonulin, the only known physiological modulator of intercellular tight junctions (69). Increased release of zonulin is associated with gut barrier dysfunction and gliadin peptides have been reported to trigger this reaction (65).

## A Role for Direct Gluten-Induced Enterocyte Damage?

**Figure 1:**
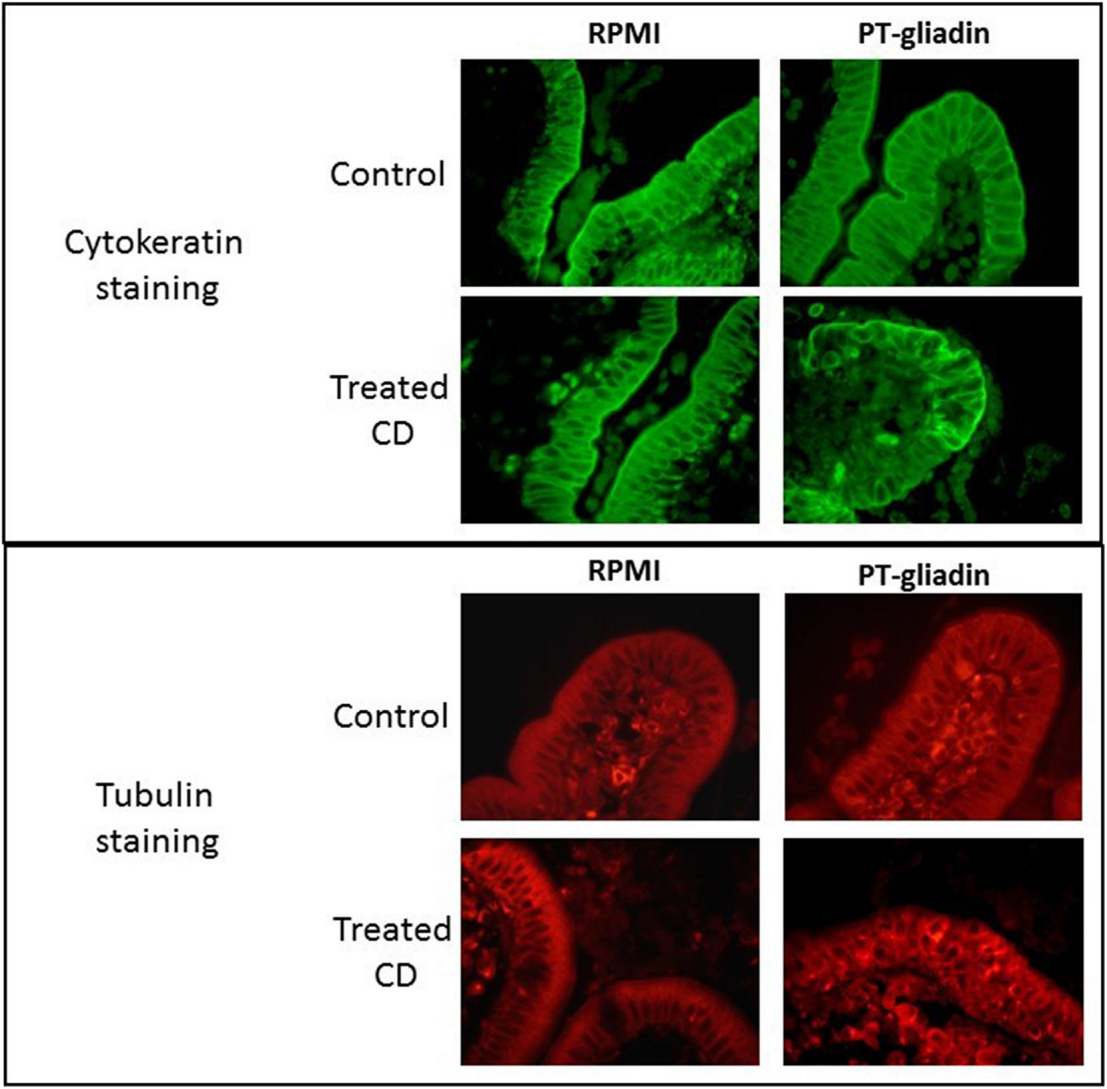
Direct effect of peptic-tryptic digests of gliadin on intestinal enterocytes. Representative images of organ culture of healthy (*n* = 5) and coeliac (*n* = 5) biopsies in the presence or absence of peptic-tryptic (PT) digests of gliadin demonstrates direct effects of gliadin. Treatment of coeliac biopsies for 24 h with PT gliadin reveals significant changes in cytokeratin and tubulin staining, as demonstrated by fluorescence microscopy.

Details of enterocyte pathology following gluten exposure have been investigated by both *in vitro* and *in vivo* challenge studies. In organ culture of biopsies taken from coeliac patients co-cultured with gluten derived proteins, evidence of rapid changes in enterocyte morphology has been reported. In several studies, gluten caused reduction in enterocyte height (70–73) and increased apoptosis of enterocytes (28, 41, 74, 75). We also performed organ culture experiments employing a peptic/tryptic digest of gluten and demonstrated derangement of several enterocyte cytoskeletal proteins, including microfilaments, intermediate filaments and microtubules; these changes were evident after 4 h of culture but were even more marked after 24 h (Figure 1) (76).

Several short-term *in vivo* challenge studies also reported evidence of rapid enterocyte damage following infusion of gluten fractions into the small intestine. When small intestinal biopsies were taken at hourly intervals, significant histological damage was observed after patients were given either gluten (77), gliadin subfractions (38, 39) or the associated wheat protein glutenin (78). The abnormalities included a reduction in enterocyte height, an increase in IELs and a reduction in the villous/crypt ratio. In some instances, these changes were noted as early as 2 h after gluten exposure (78).

Taken together, these studies demonstrate rapid changes in coeliac enterocyte morphology following gluten exposure. The mechanisms responsible have yet to be identified. Although a rapid response is more typical of innate immune involvement, there is now evidence that a histological and cytokine response to gluten and immunodominant gliadin peptides can take place within hours. A study by Fraser et al. demonstrated that *in vivo* challenge with residues 56–75 of α-gliadin resulted in reduction of enterocyte height and an increase in IELs in biopsy tissue within just 4 h (79). Moreover, it has been recently reported that oral gluten challenge causes a significant elevation of plasma IL-2, suggestive of rapid activation of T lymphocytes, again within 4 h (80). The findings of these studies support a prominent role for adaptive immunity in causing early tissue changes in the CD lesion.

## A Role for Enterocyte Proliferation?

It has been suggested that hyperproliferation of enterocytes is the principal pathological event responsible for apparent villous atrophy in CD mucosa, rather than direct destruction of the villous structure (50, 81, 82). Marsh and Heal in particular have argued this point and state that the term “villous atrophy” is a misnomer (50). They postulate that the overgrowth of the crypt cell population surrounds and dwarfs the villous structure and cause its shrunken appearance. Crypt cell proliferation was investigated by Wright et al. and an up to 6-fold increase in crypt cell production was calculated with an associated increase in the mitotic index (83).

The increased crypt cell proliferation may due to the reparative process to replace damaged enterocytes shed into the intestinal lumen. However, it has also been reported that proliferation may be due to a direct effect of gliadin peptides on the coeliac mucosa (84). In cultured coeliac duodenal biopsy tissue the p31-43 gliadin peptide was shown to increase crypt cell proliferation, apparently via the epidermal growth factor pathway (85). This effect was mediated by enhancing epithelial growth factor receptor signaling in a mechanism involving altered vesicular trafficking (84, 86). It was also postulated that this proliferative effect was further augmented by increased IL-15 production, stimulated by the gliadin peptide (29).

## A Role for Gliadin Peptides in Causing an Innate Response?

In addition to its reported effect on crypt cell proliferation (84), many additional studies have focused on the α-gliadin peptide, p31-43 and its potential to cause direct activation of the innate immune system. Within 20 min of exposure to this peptide evidence of enterocyte actin reorganization was reported in organ culture experiments (87). Further study has revealed various additional pathogenic effects. p31-43 was shown to inhibit the subunit of the chloride channel (CFTR) and thereby cause NF-kB activation, induction of IL-15 and TG2 activation, with a range of inflammatory consequences (88). Moreover, p31-43 is able to self-assemble in oligomers with potential important effects (89). *In vivo* analysis in a murine model showed that activation of the NLRP3 inflammasome, either by direct detection of oligomers or indirectly by sensing danger signals, is required for histological changes in the small intestinal mucosa (90). Finally, the peptide was shown to induce a type I interferon (IFN) response in mice (91). Of note, high levels of IFNα can be observed in the duodenal mucosa from coeliac patients (84) and this cytokine has been suggested to promote T helper (T_H_) type 1 responses in CD (92).

More recently, various studies were conducted to evaluate the role of a particular enteric virus which causes the induction of proinflammatory signals that may promote breakdown of oral tolerance to gluten (93). Commensal microbiota also has a role to play in the induction of local inflammation. Elastase-producing *Pseudomonas aeruginosa* isolated from the duodenal biopsies of CD patients was able to degrade a gluten-derived 33-mer peptide, producing shorter fragments which cross the mucosal barrier and display increased immunogenicity (94). Therefore, viral infection and the activity of *P. aeruginosa*, as examples of members of the microbiota which can elicit proinflammatory signals on intestinal cells which drive or amplify villous damage. Other mechanisms, apart from conventional immunogenic peptides, that may play a role are the amylase trypsin inhibitors present in wheat. These proteins are resistant to intestinal digestion, can directly activate Toll-like receptor 4 (TLR4) and may support intestinal T cell activation in celiac disease (95). However, the main immunogenic peptide, responsible for stimulating the adaptive immune response is a 33-mer gliadin peptide, p56-88, which contains several overlapping sequences that bind with high affinity to susceptibility HLA molecules (92). A further small eight residue gliadin peptide is reported to act through the stimulation of particular dendritic cells, can amplify the inflammatory response (96).

## A Role for Conventional and Unconventional Lymphocytes?

T lymphocytes and B cell derived plasma cells are markedly increased in the coeliac lesion. In the untreated coeliac mucosa, plasma cells secreting IgA are increased 2.4-fold and represent some 66% of antibody secreting cells (97). Increases in IgM and IgG secreting cells also are found, with these cells accounting for some 28 and 6% of plasma cells in the lesion. Antibodies of all three isotypes to gliadin and auto-antigen targets can be detected not only in intestinal secretions but also in the circulation. Assays for these antibodies are of immense value in the diagnosis and monitoring of disease activity in CD patients. In addition, gut-resident plasma cells presenting the immunodominant gluten peptide DQ2.5-glia-α1a have been shown to be abundant in the CD lesion, suggesting an important local antigen presentation role (98). These cells also act as a source of cytokines, including the chemokine CXCL10 (99).

Gluten-reactive T lymphocytes are found in the *lamina propria* and are comprised of α/β T cell receptor positive cells bearing a CD4 co-receptor, identifying them as T_H_ cells (3, 100). However, cloning studies have revealed that only 0.5–1.8% of CD gut-derived CD4 T cells are truly gluten reactive (101). These gluten-reactive cells display a T_H_1 phenotype in response to gluten peptides, with cytokine production predominated by IFNγ (102). Gliadin-specific T_H_17 cells have also been described, which co-produce IL-17 and IFNγ (103). Gluten reactive CD8^+^ T cells have also been described in the *lamina propria* following challenge with the pA2 gliadin peptide (41). Nonetheless, studies in mice and humans have shown that the presence of these gluten-reactive T cells alone is not sufficient to drive pathological changes to the villous architecture (104–106). Thus, in potential CD, where individuals demonstrate an adaptive immune response to gluten, characterized by the presence of serum endomysial antibodies, no histological lesion is present. This suggests that additional factors may be required to drive tissue damage (107).

**Figure 2:**
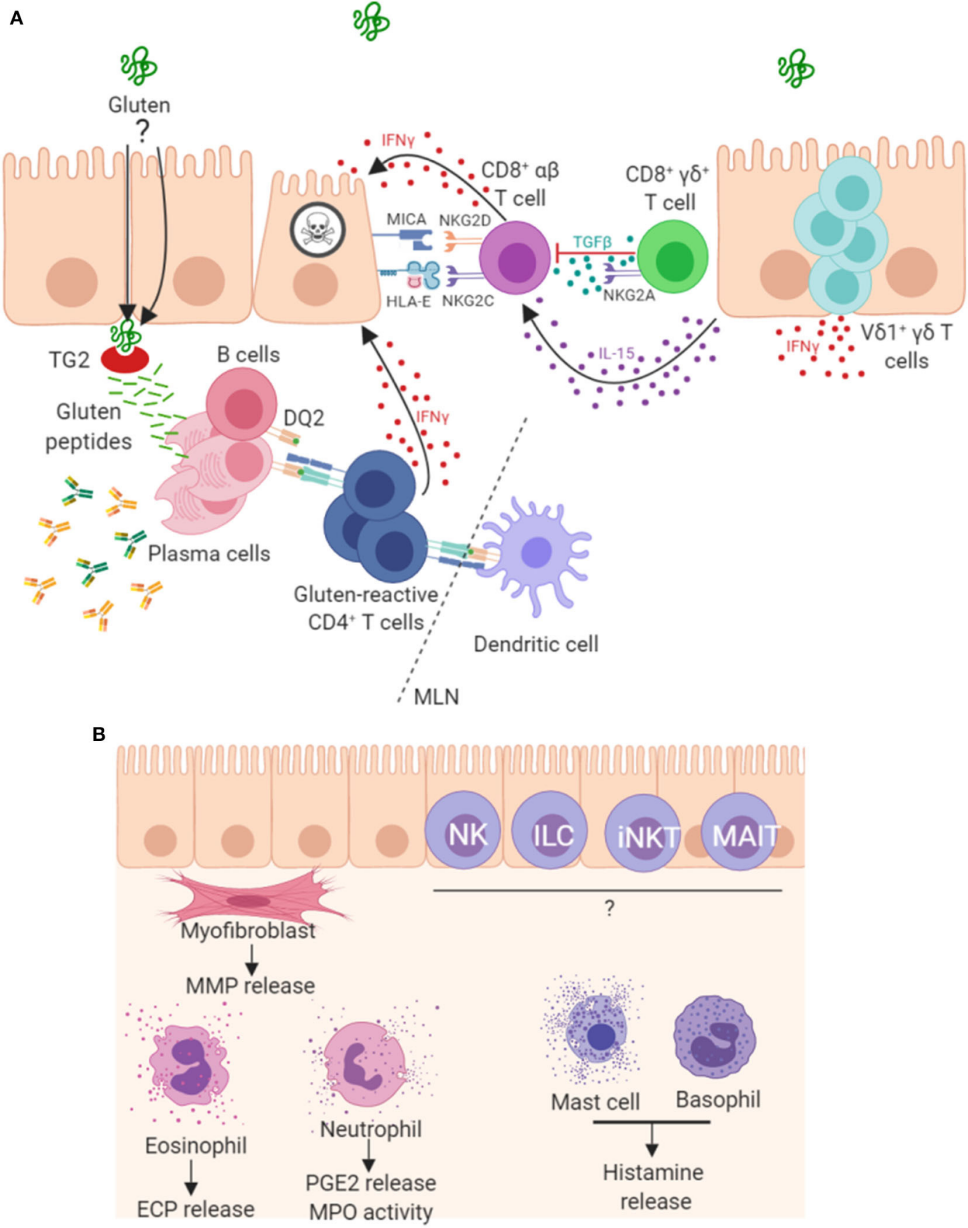
Mechanisms of pathogenesis in coeliac disease. **(A)** It is well established that peptides derived from gluten are modified by TG2 and presented by antigen presenting cells in mesenteric lymph nodes (MLN) to CD4^+^ T cells in the context of HLA-DQ2. The resulting T_H_1 type response results in IFNγ production and intestinal inflammation. Chronic inflammation leads to expansion and persistence of Vδ1^+^γδ T cells, which also contribute to IFNγ production. Gluten peptides induce expression of IL-15 and stress molecules on enterocytes. The increased levels of IL-15 promote a NK-like phenotype in CD8^+^ T cells, contributing directly to enterocyte death. A proportion of CD8^+^γδ^+^ T cells are thought to play a regulatory role through secretion of TGF-β. Plasma cells are also abundant in the lesion where many express the immunodominant gluten peptide DQ2.5-glia-α1a and are induced to secrete antibodies that bind to TG2 and other targets. **(B)** Other less well-characterized mechanisms may play a role in lesion development. Intestinal myofibroblasts contribute to tissue remodeling by the secretion of matrix metalloproteases (MMPs) and via their contractile properties. These cells strongly express TG2 and α-actin. Innate-like lymphocytes including natural killer (NK cells), innate lymphoid cells (ILC), invariant natural killer T cells (iNKT) and mucosal-associated invariant T (MAIT) cells may all contribute to the lesion. Granulocytes, including eosinophils, neutrophils and basophils, and also mast cells have been detected in higher levels and may be involved in disease pathogenesis.

Parallels have been drawn between the progression of CD and graft vs. host disease, suggesting a key role for T cells in disease pathogenesis (108). In addition to gluten-reactive CD4^+^ α/β T cells, a role for CD8^+^ α/β IELs in enterocyte destruction has also been shown, whereby these cells acquire an aberrant natural killer (NK)-like phenotype and kill enterocytes in a T cell receptor (TCR)-independent manner. These mechanisms, driven by IL-15 (46, 68), are summarized in Figure 2. This NK-like action is characterized by strong IFNγ production, upregulation of activating NK receptors NKG2D and CD94/NKG2C (46), concurrent downregulation of inhibitory co-receptors CD94/NKG2A (106) and cytotoxic ability. Engagement of activating NK receptors by stress molecules expressed on enterocytes triggers the cytolytic function of these CD8^+^ IELs. In patients with CD, enterocytes upregulate expression of stress molecules such as heat shock proteins (HSP), MHC class I polypeptide-related sequence A (MICA), HLA-E and IL-15 (45, 109, 110). However, gluten-reactive CD4^+^ T cells have been shown to be required to fully license the cytolytic NK activity of these CD8^+^ IELs (106, 111, 112). A recent mouse model of CD has also demonstrated the key role of CD4^+^ T cells and HLA-DQ8 in mediating cytotoxic lymphocyte (CTL)-driven villous destruction (111). Depletion of either CD4^+^ or CD8^+^ T cell populations prevented villous damage in these transgenic HLA-DQ8^+^ mice overexpressing IL-15. CD4^+^ T cell depletion resulted in a failure of CD8^+^ CTLs to upregulate RAE-1, the murine ligand for NKG2D, providing further evidence that the NKG2D pathway is important in CD4-mediated CTL licensing. Upregulation of QA-1, the mouse ligand for NKG2 receptors paired with CD94, was unaffected by CD4 depletion, showing that this mechanism is specific to NKG2D. This study also revealed critical roles for gluten, IL-15, HLA-DQ8, TG2, and CD4 T cells, working in concert to promote IFNγ responses and expansion of activated cytolytic CD8 IELs which mediate villous atrophy. IFNγ, a prominent cytokine in CD pathogenesis, is produced not only by gluten-reactive *lamina propria* T_H_ cells but also by populations of IELs, including γ/δ IELs (113). In addition to IL-15, cytokines IL-2 and IL-21 are two further important cytokine products of gluten reactive T_H_ cells and contribute to the adaptive immune pathogenesis of CD (80, 114–116), however, of interest they were not shown to be critical for development of villous damage in this recent mouse model (107). A study on human tissue reports that the majority of CD patients overexpress both IL-15 and IL-21 and *in vitro* analysis showed these cytokines synergise to activate CTL IEL populations and thus drive villous damage, in a cooperative and non-redundant manner (117).

Intriguingly, individuals with potential CD do not demonstrate an increase in activating NK receptors nor IL-15 or IL-21, suggesting that additional factors may also be required to drive the full NK-like phenotype. Potential candidates include gut microbial components (118) or viral infection (119, 120). This is further supported by a recent study in which ubiquitous bacterial peptides were shown to activate gliadin reactive T cells, suggesting the possibility that common bacterial antigens could act as trigger stimuli in the development of CD (121).

Whereas, CD4^+^ T cells predominate in the *lamina propria*, the human small intestinal epithelium is predominantly populated by CD8^+^ α/β IELs, γ/δ IELs, and a smaller proportion of lymphocytes which do not express a T cell receptor, and therefore are classed as innate lymphocytes (21, 122). This latter population includes NK cells and ILCs. The CD8^+^ α/β IELs group also includes mucosal-associated invariant T (MAIT) cells and, albeit at low levels, invariant NK T (iNKT) cells (23). It is assumed that most of these cell types play a lesser but similar destructive role to their conventional T cell counterparts, since unconventional T cells constitutively express NK markers, display an effector memory phenotype, and are capable of rapid and potent cytolytic responses. Indeed, ILCs have been shown to be capable of killing enterocytes via the NK receptor DNAM1 (123).

The role of discrete IEL subsets warrants further study, particularly in light of the long-held observation that γ/δ IELs remain elevated in the coeliac gut long after removal of gluten from the diet and resolution of intestinal damage (23, 124). In contrast to the deleterious role proposed for CD8^+^ α/β IELs in CD, it is hypothesized that γ/δ IELs play a more regulatory role in the gut (125, 126). We and others have described an abundance of Vδ1 type γ/δ T cells in the CD epithelium, in both pediatric and adult CD (23, 127–130). This subset is known to possess potent cytolytic and regulatory functions in humans (131). Like their mouse counterparts, human γ/δ IELs in skin can secrete growth factors, specifically insulin-like growth factor, and play an important role in tissue repair (132). Whether this active role in tissue repair also occurs in the gut is unclear, but human NKG2A^+^ CD8^+^ γ/δ IELs have been shown to effectively dampen the proinflammatory and cytotoxic action of their α/β IELs counterparts via production of the immunosuppressive cytokine TGF-β (133). TGF-β is an immunosuppressive cytokine which exerts many anti-inflammatory effects, including driving differentiation of regulatory T cells and T_H_17 cell populations, which then produce more TGF-β in an autocrine manner (134).

This suggests that lymphocyte-mediated damage to the coeliac small intestine may require dysregulation of both α/β and γ/δ IEL subtypes. In this scenario, a two-step process would be required to mediate gut damage—CD8^+^ α/β IELs acquire an aberrant NK-like cytotoxic phenotype coupled with γ/δ IELs losing regulatory function. This raises an intriguing possibility that maintenance of γ/δ T cell regulatory function could explain the phenotype of potential CD, a scenario where γ/δ IELs keep α/β IEL cytotoxicity in check. Indeed, recent studies detailing long-term genomic and functional changes in the composition of the γ/δ IEL compartment in CD has shown that a subset of Vγ4^+^/Vδ1^+^ type γ/δ IELs, which have a role in tissue healing and homeostasis, is lost and replaced by a persistent IFNγ producing Vδ1^+^ T cell population, thereby supporting this hypothesis (21, 22). The role of other lymphocyte subsets such as MAIT cells, iNKT cells and NK cells in CD is less well-understood (23). NK cells in particular appear to be capable of both deleterious and protective effects on the intestine and are also susceptible to functional and metabolic inhibition by TGF-β (135); thus, their contribution to CD pathogenesis requires further elucidation (136).

## A Role for Innate Immune Cells in the Intestinal Lesion?

In addition to the increased number of T cells and plasma cells in patients with active CD, several studies describe increased populations of cells of the innate immune response, including eosinophils (32–34), basophils (50), mast cells (32, 35, 36), neutrophils (137, 138) and dendritic cells (139, 140). The potential contribution of both eosinophils and mast cells is supported by experiments involving gliadin challenge to an isolated segment of jejunum: this caused a four-fold increase in eosinophil granule specific protein secretion and a two-fold increase of histamine secretion, with maximum levels found within 1 h (141). A prominent extracellular deposit of eosinophil granule specific protein in the *lamina propria* of the atrophic intestinal mucosa was also found (31). Furthermore, eosinophils were noted to be in an activated state in CD and Brandtzaeg postulated that IgA might play a role in both eosinophil recruitment and activation (97). Mast cell numbers are also increased in active CD, and found to correlate with the Marsh histological score and become cellular sources of TNFα, IL-6, IL-17 and monocyte chemoattractant protein 1 (36, 50). These data indicate that eosinophils and mast cells may both be involved in early gliadin-induced reactions in the small intestine and could contribute to the celiac lesion.

Neutrophils may also play a role in the coeliac lesion; in the early phase of gluten challenge, increased numbers of these cells have been observed with a 20-fold increase calculated (137, 138). The rapid production of the chemokine IL-8 by gluten activated T cells helps explain this neutrophil migration (80). In isolated jejunal segment experiments, gluten exposure caused a 5-fold increase in prostaglandin E2 (142) and a 3.5-fold increase in myeloperoxidase in the perfusion fluid (31). Furthermore, using gene expression profiling, chronic recruitment of activated neutrophils to CD biopsy tissue was discovered, even in patients in remission (143). In another study, after a 3-day gluten challenge, an increase in density of neutrophils as well as a rapid accumulation of monocyte/dendritic cells was observed (140). Of interest, in a murine study, gliadin peptides were found to have neutrophil chemoattractant properties (144). Dendritic cells are the critical players in innate immunity as well as adaptive response. Distinct subsets may display different functions, as induction of strong inflammatory response, driving the gluten-specific T cell response and control the immune response by inducing regulatory T cells (139, 145).

## A Role for Anti-TG2 Antibodies in CD Pathogenesis?

Detection of anti-TG2 autoantibodies is an exceptionally specific and sensitive tool used for CD diagnosis. The most commonly accepted model for the development of this autoantibody response is the hapten-carrier complex mechanism, as proposed by Sollid et al. (146). The hypothesis suggests that TG2-gliadin complexes are presented by TG2-specific B cells to gliadin-specific T cells and receive help for antibody production. While this model does not necessitate TG2-reactive T cells, separate studies by Comerford et al. and Ciccocioppo et al. demonstrate that these autoreactive T cells can be detected in patients (147, 148).

Whether or not autoantibodies play a role in the development of the lesion remains unclear. The fact that IgA deficiency does not preclude the development of CD suggests that IgA isotype autoantibodies are not essential for disease development. However, in the related gluten-sensitive condition dermatitis herpetiformis, it appears that autoantibodies against transglutaminase 3 (another member of the TG family) do appear to play a role in pathogenesis as demonstrated by the presence of IgA deposits at sites of neutrophil infiltration in the skin (149). Unlike CD, dermatitis herpetiformis is not observed in patients that are IgA deficient (150). It has been proposed that anti-TG2 autoantibodies influence the disease process in CD by having a direct effect upon enterocytes. Purified anti-TG2 antibodies have been shown to inhibit crypt cell differentiation (151), interfere with proliferation by binding membrane TG2 (85), and enhance gliadin trafficking across the gut epithelium (152). It has also been suggested that anti-TG2 could interfere with enterocyte differentiation by blocking TGF-β activation, a cytokine that plays an important role in this process (151). Other effects have been reported including inhibition of angiogenesis, and increases in vascular permeability (153).

## A Role for Complement?

Few studies have examined the possibility of complement involvement in CD pathogenesis. In early reports, C3 deposits and proteins of the terminal complement pathway were shown in the small intestine, concentrated sub-epithelially and in the *lamina propria* (37). Untreated CD patients typically have high levels of IgG1 and IgG3 anti-gliadin antibodies in their serum (154) both of which are capable of activating complement (155). Sub-epithelial IgA-TG2 deposits, found in the early stages of CD (156) might also play a role, and polymeric IgA has been shown to activate complement via the MBL pathway (157). Activation of the classical complement pathway would result in increased production of C3a and C5a, both capable of contributing to the coeliac lesion by increasing vascular permeability and causing mast cell degranulation. In addition, C5a as a chemotactic factor could increase the migration of eosinophils, neutrophils and monocytes to the lesion and initiate release of products such as prostaglandins (142). Complement activation could therefore explain the rapid onset of gluten induced symptoms observed in some patients with CD (11, 12).

## A Role for Intestinal Myofibroblasts?

Intestinal subepithelial myofibroblasts possess a broad range of biological functions and are likely to play a central role in architectural remodeling in CD. Myofibroblasts synthesize many components required for the extracellular matrix and the basement membrane and also control the degradation of these structures through the release of matrix metalloproteases (MMPs) along with inhibitors of these enzymes, the tissue inhibitors of metalloproteases (TIMPs) (158). Several studies have described increased mRNA and protein levels of MMP-1, MMP-3, MMP-9, MMP-12, and TIMP-1 in the coeliac lesion (159–161). In some instances, levels of MMPs correlated with the degree of histological damage. Several cytokines are critical to the function of myofibroblasts, including TGF-β (162). In inflammatory bowel disease the production of MMPs by myofibroblasts in is thought to be driven by IL-1β and TNF-α (163), and the latter cytokine is produced by IEL in CD (164). IFNγ and IL-21 are additional candidate cytokines which may stimulate MMP production in the coeliac lesion (159, 165).

**Figure 3:**
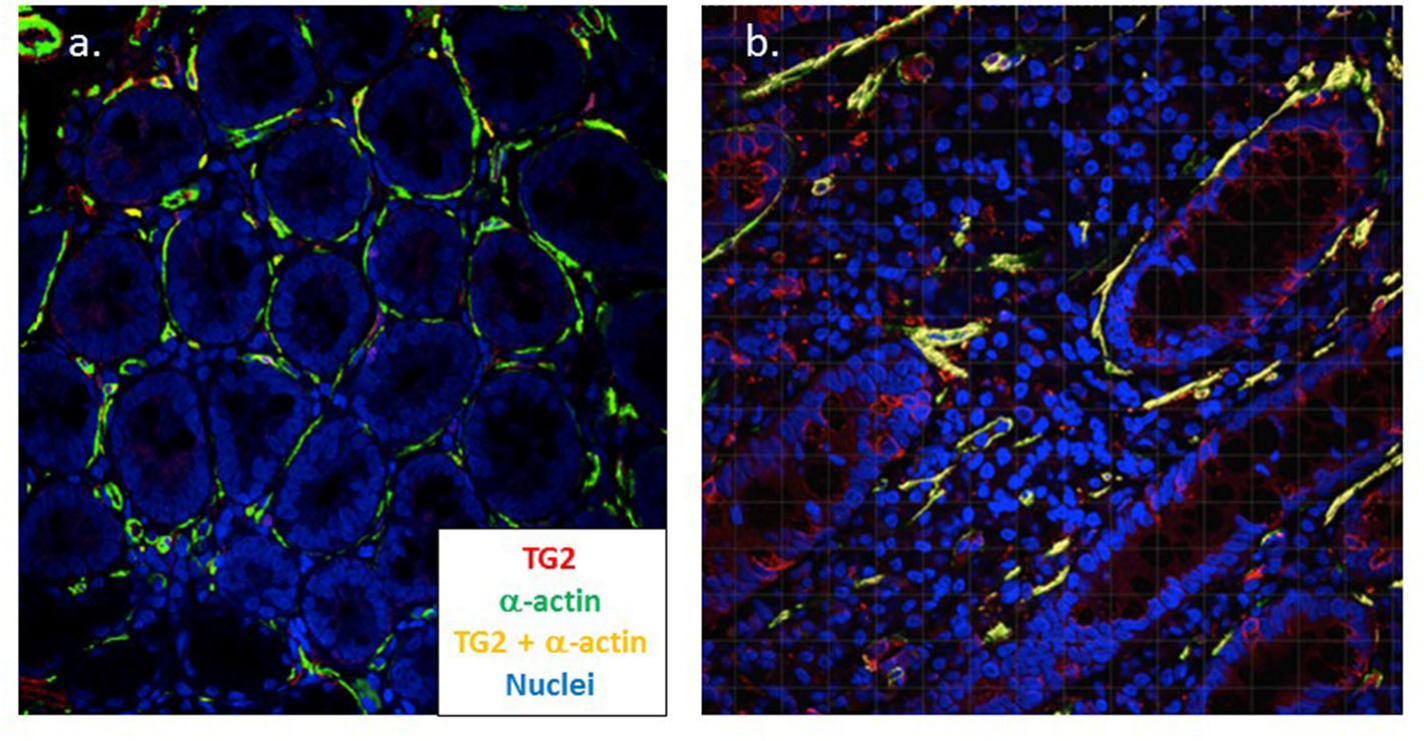
Myofibroblasts strongly co-express TG2 and α-actin in coeliac disease. Dual color confocal microscopy demonstrates that intestinal myofibroblasts stain positive for α-actin (green) in healthy control tissue (*n* = 5) **(a)**. In active coeliac disease (*n* = 11) **(b)** these cells upregulate TG2 (red) and significant co-expression is apparent (yellow) (Cooper et al., manuscript in preparation). Original magnification x40.

In assembling the structure of the small intestine, TG2 plays a central role and myofibroblasts have been shown to strongly express TG2 in active CD (166). Using confocal microscopy, we have confirmed this finding and demonstrated that TG2 expression strongly co-localizes with increased smooth muscle α-actin expressed by these cells in active disease (manuscript in preparation) (Figure 3). In an *in vitro* model, it was found that IgA autoantibodies to TG2 interfere with the effect of TGF-β on myofibroblasts; this resulted in the increased proliferation of enterocytes (151). Finally, myofibroblasts also interact with the immune system, express MHC class II as well as CD80 and CD86, and have been shown to act as non-professional antigen presenting cells (167). It has also been demonstrated that myofibroblasts induce the proliferation and differentiation of regulatory T cells (168) suggesting a possible role in immune homeostasis.

Taken together, these findings suggest that intestinal myofibroblasts could play an important tissue remodeling role in the coeliac lesion as well as being a potential venue for epitope spreading. The potential contribution of myofibroblasts with multiple cellular and other immune components to CD pathogenesis is represented in Figure 2B.

## Conclusion

Features of CD suggest that it can be considered an autoimmune disease with gluten as an environmental trigger causing activation of a highly specific adaptive immune response. An increase in IELs is a classic finding in CD and some conventional lymphocytes with a NK-like phenotype contribute to enterocyte destruction. The function of other IEL populations, such as γ/δ IELs, in CD is less certain and work to date suggests these cells may play an important local immune regulatory role. Intestinal epithelial cells, through their production of IL-15, play a dynamic role in disease pathogenesis in addition to being targets of the immune response. There is also evidence that cells of the innate immune system, including eosinophils, mast cells and neutrophils, contribute to disease pathogenesis. A further cell population, myofibroblasts, are an important source of TG2 and metalloproteases and therefore may also play a central pathogenic role in CD. Controversy surrounds the issue of whether non-immune gliadin peptides contribute to the disease process. Some studies report that one such peptide, p31-43, can cause direct damage to enterocytes and also stimulate enterocyte proliferation. The failure to identify a receptor for this peptide has been used to reject its involvement in the disease process. If alternate gluten peptides cause innate cell activation, this will be important in designing future gluten avoidance strategies.

## Author Contributions

All authors contributed to the planning, writing and editing of this manuscript, and approve this submitted version for publication. MD and GB contributed equally to creating the manuscript. Figures were constructed by GB and MD.

## Conflict of Interest

The authors declare that the research was conducted in the absence of any commercial or financial relationships that could be construed as a potential conflict of interest.
